# Protein Phosphorylation and Redox Modification in Stomatal Guard Cells

**DOI:** 10.3389/fphys.2016.00026

**Published:** 2016-02-05

**Authors:** Kelly M. Balmant, Tong Zhang, Sixue Chen

**Affiliations:** ^1^Department of Biology, Genetics Institute, University of FloridaGainesville, FL, USA; ^2^Plant Molecular and Cellular Biology Program, University of FloridaGainesville, FL, USA; ^3^Proteomics and Mass Spectrometry, Interdisciplinary Center for Biotechnology Research, University of FloridaGainesville, FL, USA

**Keywords:** phosphorylation, redox, guard cell, signaling, abiotic and biotic stresses

## Abstract

Post-translational modification (PTM) is recognized as a major process accounting for protein structural variation, functional diversity, and the dynamics and complexity of the proteome. Since PTMs can change the structure and function of proteins, they are essential to coordinate signaling networks and to regulate important physiological processes in eukaryotes. Plants are constantly challenged by both biotic and abiotic stresses that reduce productivity, causing economic losses in crops. The plant responses involve complex physiological, cellular, and molecular processes, with stomatal movement as one of the earliest responses. In order to activate such a rapid response, stomatal guard cells employ cellular PTMs of key protein players in the signaling pathways to regulate the opening and closure of the stomatal pores. Here we discuss two major types of PTMs, protein phosphorylation and redox modification that play essential roles in stomatal movement under stress conditions. We present an overview of PTMs that occur in stomatal guard cells, especially the methods and technologies, and their applications in PTM identification and quantification. Our focus is on PTMs that modify molecular components in guard cell signaling at the stages of signal perception, second messenger production, as well as downstream signaling events and output. Improved understanding of guard cell signaling will enable generation of crops with enhanced stress tolerance, and increased yield and bioenergy through biotechnology and molecular breeding.

## Introduction

Stomata are composed of a pair of specialized epidermal cells termed guard cells, which are responsible for regulating gas exchange and water loss through changing the size of the stomatal pores. The opening and closing of stomatal pores are affected by numerous factors, such as humidity, CO_2_, temperature, light, hormones, and pathogens. Changes in the turgor and volume of guard cells accordingly are required for stomatal movement, which are controlled by complex signaling networks (Azoulay-Shemer et al., 2015).

Abscisic acid (ABA) plays important roles in a broad range of plant physiological processes (e.g., seed germination and seedling growth) and plant responses to abiotic and biotic stresses (Lee and Luan, 2012). Under high salinity and drought conditions, the increased levels of ABA are perceived by the guard cells to promote stomatal closure and to inhibit of stomatal opening (Assmann, 2003). The mechanisms underlying ABA signaling in guard cells have been extensively studied (Pei et al., 1997; Schroeder et al., 2001; Assmann, 2003; Acharya et al., 2013; Zhang et al., 2015), which involve the binding of ABA to the receptors, activation of protein kinases, production of second messengers such as reactive oxygen species (ROS) and nitric oxide (NO), regulation of membrane ion channels, and eventually the decrease in turgor and stomatal closure (Schroeder et al., 2001; Zhang et al., 2015). In addition to abiotic stress, guard cells play an important role in limiting pathogen entrance to the plant body. The guard cell response to bacteria is triggered by the recognition of pathogen associated molecular patterns (PAMPs) by pattern recognition receptors (PRRs) on the plasma membrane. Upon PAMP recognition, one of the earliest responses is the change in ion fluxes across the membrane, leading to a rapid and transient extracellular alkalization and increase of Ca^2+^ in the cytosol (Boller and Felix, 2009). Ca^2+^ functions as a second messenger, activating downstream signaling players such as calcium-dependent protein kinases (CDPKs) to promote stomatal immunity responses. In addition, the apoplastic production of ROS by NADPH oxidase (Boller and Felix, 2009) is a hallmark of successful recognition of plant pathogens. Subsequent plant immune responses include transcriptional reprogramming, which involves the regulation of ROS homeostasis and activation of other protein kinases such as mitogen-activated protein kinases (MAPKs) (Boudsocq et al., 2010).

Stomatal studies are technically challenging because guard cells are small and of low abundance in leaves (Tallman, 2006). Methods for isolating guard cell protoplasts with relatively high purity have been reported over the past 30 years (Outlaw et al., 1981; Gotow et al., 1982, 1984; Zhu et al., 2010, 2014; Obulareddy et al., 2013). They have contributed considerably to the understanding of guard cell signaling. However, these methods are usually laborious and the yield is relatively low. The general principle of guard cell isolation is to release the guard cells from epidermal peels in a two-step process. In the first step the pavement and mesophyll cells are removed, and in the second step the guard cell wall is digested to facilitate the release of the guard cell protoplasts. It is important to note that there are important variations of the procedures according to different plant species (Zhu et al., 2016).

Stomatal movement in response to abiotic and biotic stresses is a fast process, which requires an efficient molecular regulation mechanism to relay the signals. Phosphorylation and redox control of the key players during both the signal perception and transduction in plant responses to abiotic and biotic stresses have demonstrated the high efficiency of protein PTMs in cell signaling (Grennan, 2007; Waszczak et al., 2015; Zhang et al., 2015). As the relevance of PTMs in plant stress responses has been demonstrated by independent studies over the years (Kodama et al., 2009; Lindermayr et al., 2010; Stecker et al., 2014; Kim et al., 2015; Yang et al., 2015), there is a growing interest to understand how specific PTMs control various aspects of stomatal guard cell functions. In this review, the frequently used approaches and methods in identification and quantification of PTMs are described. The main objective is to focus on the phosphorylation and redox events, and the recently identified proteins that undergo PTMs in guard cells in response to phytohormone and stress signals. We also discuss the different types of PTMs in the regulation of stomatal movement, and the challenges and perspectives of PTM proteomics.

## Advances in protein PTM technologies

### Significance of PTMs in biological processes

PTMs include chemical modifications of specific amino acid residues of a protein and/or cleavage of the translated sequence. They greatly increase the structural and functional diversity of proteins in a proteome. Currently, more than 300 different types of PTMs have been identified (Zhao and Jensen, 2009), including phosphorylation, glycosylation, acetylation, nitrosylation, ubiquitination, and proteolytic cleavage. These modifications affect the properties of the proteins (e.g., charge status and conformation), resulting in changes of activity, binding affinity, localization as well as stability. Most PTMs are highly controlled in the cells, and they often serve as rapid, specific, and reversible molecular switches to regulate biochemical and physiological processes. Different PTMs have also been shown to crosstalk in the modulation of molecular interactions between proteins or regulation within the same protein through multiple site modification, e.g., the histone code (Bannister and Kouzarides, 2011). Therefore, identification and functional characterization of PTMs are critical toward deciphering their roles in cellular processes in many different areas of biology and biomedical research.

### Qualitative analysis of PTMs

In the past, PTMs were often studied at a specific amino acid residue of a particular protein level using molecular and biochemical approaches (Zhu et al., 2000; Reimer et al., 2002). Nowadays, the advances in biological mass spectrometry (MS) have allowed accurate identification and quantification of PTMs at the proteome scale. Two-dimensional gel electrophoresis (2-DE) was widely used in the early years of proteomics to identify PTMs, such as phosphorylation, nitrosylation, acetylation, and glycosylation (Llop et al., 2007; Roux et al., 2008; Scheving et al., 2012). Because PTMs can alter the isoelectric point and/or molecular weight, they may be detected when a change of spot location on the gel is observed between different samples. Different PTM protein stains have been developed to reveal specific PTMs, such as ProQ diamond and ProQ emerald to detect phosphoproteins and glycoproteins in the gels, respectively (Steinberg et al., 2001; Schulenberg et al., 2003; Ge et al., 2004). A big challenge has been to identify the PTM peptides and map the sites of modifications due to the low abundance nature of the modified protein species.

To overcome the challenge of capturing the relatively low abundance of PTM proteins compared with unmodified proteins, fractionation, and/or enrichment strategies have been employed during sample preparation (Lenman et al., 2008; Guo et al., 2014a; Aryal et al., 2015). The MS-based proteomics coupled with PTM enrichment typically has four steps. First, samples containing the total protein of interest are digested by a protease, such as trypsin. Second, the resulting peptides are subject to enrichment, in order to separate the PTM peptides of interest from the often abundant non-modified peptides. Third, the isolated PTM-peptide is analyzed by liquid chromatography (LC)-MS/MS for peptide identification and PTM site mapping. Finally, the MS spectra of the peptides are analyzed using different software algorithms and/or evaluated manually to ensure the accuracy and statistical significance of the data.

Among the different fractionation and enrichment strategies, affinity-based approaches are commonly used to enrich PTM proteins/peptides (Blagoev et al., 2004; Rush et al., 2005; Zhang et al., 2005; Fíla and Honys, 2012; Wang et al., 2015b). The affinity-based enrichment has the advantage of relatively high specificity and significant reduction of sample complexity for downstream LC-MS/MS analyses. For example, antiphosphotyrosine antibodies were successfully used to enrich for peptides with phosphotyrosines residues (Blagoev et al., 2004; Rush et al., 2005; Zhang et al., 2005). However, the antibody-based method is often limited by the availability and quality of the antibodies for the specific PTM of interest. Thus, in order to overcome this limitation, several non-antibody based strategies have been developed. For instance, immobilized metal affinity chromatography (IMAC) utilizes a metal chelating agent to bind trivalent metal cation, such as Fe^3+^ or Ga^3+^ (Thingholm and Jensen, 2009). The charged resin is used to bind phosphoproteins or phosphopeptides. Although this strategy is widely used, it has the following shortcomings: (1) If multiply phosphorylated peptides are present in high abundance, they may saturate the IMAC resin, resulting in retention of few singly and doubly phosphorylated species (Thingholm et al., 2008). (2) Acidic peptides will be enriched along with the phosphopeptides (Thingholm et al., 2008). In order to overcome this issue, the incubation buffer needs to be acidified to pH 2–2.5. At this pH, most acidic amino acids will be protonated, which will mask the negative charge of the carboxyl groups, preventing acidic peptides from binding onto the column. In contrast, at this pH most of the phosphate moieties are deprotonated and will bind to the column (Fíla and Honys, 2012). Another approach is to use titanium dioxide (TiO_2_) as a substitute for the metal chelating resin. The use of TiO_2_ resin under acidic conditions also prevents the retention of acidic peptides (Fíla and Honys, 2012). Interestingly, these two approaches are complementary in that IMAC has higher affinity for multiply phosphorylated peptides, while TiO_2_ preferentially binds singly phosphopeptides (Silva-Sanchez et al., 2015). Therefore, application of both approaches in a single experiment leads to a high coverage of the phosphoproteome.

For cysteine redox modifications, such as S-nitrosylation, a classic biotin-switch method developed by Jaffrey et al. (2001) was often used. Free cysteines of proteins are firstly blocked by a thiol-reactive reagent through alkylation. The S-nitrosylated cysteines are then reduced using ascorbate, which is not a strong reducing reagent allowing specific reduction of the S-NO bonds. After chemical substitution with a biotin-containing affinity molecule, *N*-[6-(biotinamido)hexyl]-3′-(2′-pyridyldithio) propionamide (biotin-HPDP), the biotinylated proteins/peptides can be enriched by avidin chromatography. Although the classic biotin-switch method has been widely used, and over 300 proteins have been reported to be S-nitrosylated using this method (Lefièvre et al., 2007; Forrester et al., 2009), there are some technical issues inherent to this approach. The disulfide bonds in the proteins may decrease the efficiency of trypsin digestion and further peptide identification (Imai and Yau, 2013). Furthermore, the decomposition of biotin-HPDP may lead to a side reaction with free thiols, which can introduce false-positive signals through disulfide interchange (Forrester et al., 2007). Alternatively, a Thiopropyl Sepharose 6B (TPS6b) enrichment method was developed. The free thiols are alkylated during protein extraction. The proteins are then digested and further reduced prior to enrichment. TPS6b captures reduced thiols via disulfide exchange. TPS6b was initially used to increase the depth of proteome coverage for discovery experiments (Tambor et al., 2012). To date, it has been applied in several redox proteomics studies using cyanobacteria (Guo et al., 2014b), rat myocardium (Paulech et al., 2013), at enrichment efficiencies >95%. Recently, a six-plex iodoTMT technology has been developed to identify and quantify redox cysteines, including S-nitrosylation. Similar to the biotin-switch, free thiols are labeled with iodoTMT, and the TMT-labeled proteins or peptides can be enriched using an anti-TMT resin. This technology allows analysis of up to six samples simultaneously, thus increases throughput and reproducibility.

### Quantitative analysis of PTMs

Multiple proteomics tools are available to quantify the absolute or relative abundances of proteins and their specific PTMs. The quantification of PTMs is crucial, since simple identification of a modification may not provide adequate information for determining its functional importance. *In vivo* and *in vitro* labeling methods have been developed to couple with MS in order to identify, map, and quantify PTMs (Gygi et al., 1999; Goodlett et al., 2001; Ong et al., 2002; Ross et al., 2004; Balmant et al., 2015; Glibert et al., 2015; Parker et al., 2015). Stable isotopes can be used to label proteins *in vivo* via metabolic incorporation. In this approach, one set of sample is grown in a natural nitrogen source (N^14^) and the other set is grown in a substituted isotopic nitrogen source (N^15^) as either an amino acid (stable isotopic labeling of amino acids in cell culture, SILAC) or an inorganic nitrogen source (K^15^NO_3_) (Thelen and Peck, 2007; Stecker et al., 2014; Minkoff et al., 2015). In SILAC, since the isotopes are introduced as a specific amino acid, the mass differences between the heavy and light peptides in the MS scan can be predicted, making the quantification easy. However, this approach is challenging in plant studies, since plants can synthesize amino acids from inorganic nitrogen. For example, the labeling efficiency achieved using exogenous amino acid in *Arabidopsis* cell cultures has been reported to only 70–80% (Gruhler et al., 2005). In contrast, metabolic labeling with ^15^N as a inorganic source has been shown to achieve 98% incorporation in both intact plants (Ippel et al., 2004) and cell cultures (Engelsberger et al., 2006). However, the mass difference between differentially labeled samples cannot be easily predicted. Sophisticated software is needed to perform quantitative analysis, which can be challenging when working with highly complex samples (Thelen and Peck, 2007).

Alternatively, isotope labeling can be done to extracted proteins/peptides *in vitro* through several different approaches, e.g., isotope-coded affinity tag (ICAT), isobaric tag for relative and absolute quantification (iTRAQ), tandem mass tag (TMT), and iodoTMT. Except for ICAT, the relative quantification of peptides between samples is obtained by comparing the ion intensities of the different tags in the MS/MS spectra. The use of stable isotope labeling for absolute quantification requires internal standards, which are pre-selected synthetic peptides with isotope amino acids from a protein of interest. An absolute quantification of a PTM can be achieved by measuring the abundances of the modified and unmodified peptides and comparing them with the known amount of the isotope standard used (Xie et al., 2011). Recently, the use of label-free approaches to quantify PTMs has shown promise. Label-free analysis allows direct comparison of MS signals between any numbers of samples, which makes it applicable to any types of samples, avoiding isotope reagent costs. One label-free approach is spectral counting, where the levels of a modified form of a protein can be estimated by counting the number of the MS/MS spectra of the modified peptide from the protein. It has been noted that the number of assigned MS/MS spectra directly correlates with protein amount (Cooper et al., 2010; Olinares et al., 2011). Although spectral counting is fairly reliable in the measurement of large changes, its accuracy decreases considerably when measuring small changes of proteins (Jurisica et al., 2007) This is why peptide precursor peak alignment and peak area based label-free approach has been more popular in accuracy and robustness (Zhu et al., 2009; Zhang et al., 2010; Lin et al., 2014b).

It is important to note that although all the approaches mentioned above have found utility in the identification and quantification of PTMs, they do not often address the issue of protein turnover in the course of the experiment. Overlooking this important issue may lead to misleading results (Muthuramalingam et al., 2013; Go et al., 2014). In order to account for differences in global protein level change, which could lead to a false positive or false negative result, researches have started to acquire PTM proteomics results and total protein proteomics results from parallel or different studies (Rose et al., 2012; Zhu et al., 2014). However, the success of this strategy is often low because some proteins identified in the PTM proteomics experiments are either absent or not quantified with confidence in the total proteomics experiments (vice versa) due to experimental variation and MS2 stochastical sampling (Chong et al., 2006; Lee and Koh, 2011). To overcome this problem, Parker et al. (2015) developed a double-labeling strategy, called cysTMTRAQ, where the isobaric tags iTRAQ and cysTMT are employed in a single experiment for the simultaneous determination of quantifiable cysteine redox changes and protein level changes. This notion of normalizing against total protein turnover can certainly be applied in the studies of other PTMs. PTMs exist in many different forms, are highly dynamic and important in rapid adjustment of protein functions as molecular switches (Lothrop et al., 2013). The aforementioned approaches and the development of new tools are expected to advance the PTMs studies in many areas of biology.

## Protein phosphorylation in stomatal functions

Protein phosphorylation provides plants with a rapid and versatile mechanism to allow guard cells to respond rapidly to different environmental changes and adjust stomatal aperture accordingly (Zhang et al., 2015; Zou et al., 2015). Although the involvement of protein kinases and phosphorylation in stomatal movement has been known for decades, detailed molecular mechanisms connecting the key components have just emerged during the past 5 years. For instance, blue-light triggered stomatal opening is featured with phosphorylation and activation of the plasma membrane H^+^-ATPase by Blue Light Signaling 1 (BLUS1) (Takemiya et al., 2013). Here we focus on recent progress on the functions of protein phosphorylation in stomatal movement under abiotic and biotic stresses.

### Protein phosphorylation in guard cells under abiotic stresses

**Figure 1:**
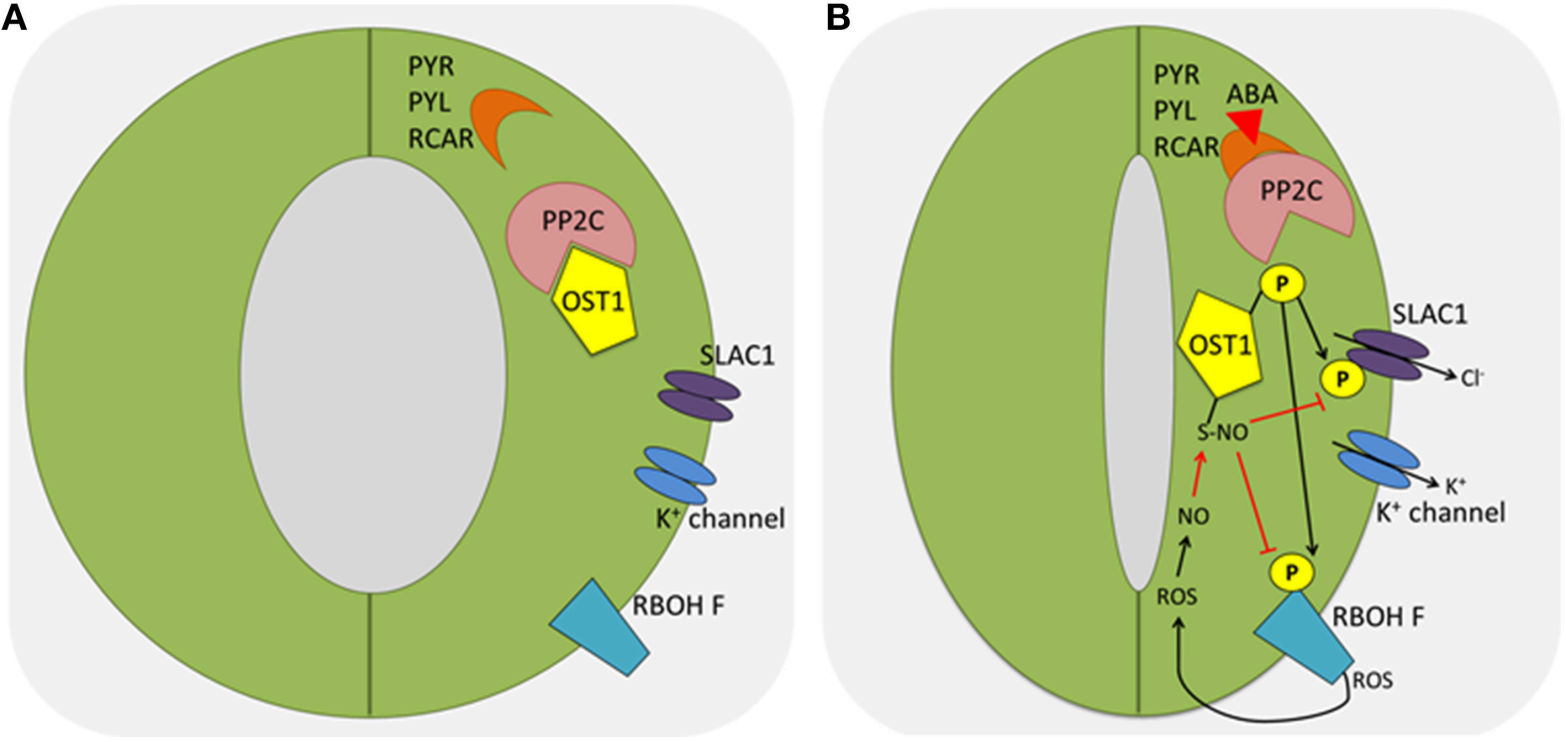
**Protein phosphorylation and redox modification in stomatal closure triggered by ABA**. **(A)** In the absence of ABA, type A PP2C inhibits the kinase activity of OST1. **(B)** In the presence of ABA, ABA binds to its receptor PYR/PYL/RCAR, which further binds and inhibits PP2C, releasing and activating OST1. Activated OST1 phosphorylates an array of substrates, including RBOH F and SLAC1. Phosphorylated and active RBOH F promotes ROS burst. Later, ROS can activate Ca^2+^ spikes in the cytosol, which can be further transduced by CDPK and CIPKs via phosphorylation of downstream target proteins. In addition, ROS can modify OST1 and RBOH to inhibit their activities as a feedback mechanism to tune down ABA signaling (red arrows). ABA, abscisic acid; PP2C, protein phosphatase 2C; OST1, Open Stomata 1; PYR, pyrabactin resistance; PYL, PYR like; RCAR, regulatory components of ABA receptors; SLAC1, slow anion channel-associated 1; ROS, reactive oxygen species; NO, nitric oxide; RBOH F, respiratory burst oxidase protein F; CDPK, calcium dependent protein kinase; CIPK, CBL (Calcineurin B-like)-interacting protein kinase.

Guard cells are responsive to a plethora of environmental factors. The drought stress induced ABA signaling pathway has been well studied. In guard cells, the central node in the core ABA network is the Sucrose non-fermenting Receptor Kinase 2.6 (SnRK2.6), also known as Open Stomata 1 (OST1). In the absence of ABA, type A Protein Phosphatase 2C (PP2C) inhibits the kinase activity of OST1. In the presence of ABA, ABA binds to its receptor PYRabactin resistance/ PYrabactin-Like/Regulatory Components of ABA Receptor (PYR/PYL/RCAR). This hormone-receptor complex further binds and inhibits PP2C, thus releasing OST1 (Geiger et al., 2011; Lee et al., 2013; Zhang et al., 2015). Activated OST1 phosphorylates an array of substrates (Figure 1), including Respiratory Burst Oxidase Homolog (RBOH F) (Sirichandra et al., 2009; Acharya et al., 2013), SLow Anion Channel-associated 1 (SLAC1) (Vahisalu et al., 2008), QUickly-activating Anion Channel 1 (QUAC1) (Imes et al., 2013), K^+^ inward rectifying channel (KAT1) (Sato et al., 2009; Takahashi et al., 2013), and membrane water channel Plasma membrane Intrinsic Protein 2;1 (PIP2;1) (Grondin et al., 2015, Table 1). Phosphorylation of the substrates leads to the ROS burst, the promotion of anion and water efflux, and the inhibition of K^+^ influx. ROS can activate Ca^2+^ spikes in the cytosol, which can be further transduced by CDPK and CIPKs via phosphorylation of downstream target proteins (Drerup et al., 2013; Ye et al., 2013). Genetic and biochemical data indicated that MAPKs and some CDPKs such as CPK8 are activated by ABA downstream of ROS production in guard cells (Jammes et al., 2009; Wang et al., 2011; Marais et al., 2014; Zhang et al., 2015; Zou et al., 2015).

**Table 1:** **List of proteins undergoing PTMs in plant response to abiotic and biotic stresses**.

**Organism**	**PTM**	**Modifier**	**Target**	**Evidence in GC**	**References**
*A. thaliana*	Phosphorylation	OST1 protein kinase	AtRBOH F	Yes	Sirichandra et al., 2009
*A. thaliana*	Phosphorylation	OST1 protein kinase	SLAC1	Yes	Maierhofer et al., 2014
*A. thaliana*	Phosphorylation	CPK6	SLAC1	Yes	Brandt et al., 2012
*A. thaliana*	Phosphorylation	CPK21/23	SLAC1	Yes	Geiger et al., 2010
*A. thaliana*	Phosphorylation	OST1 protein kinase	QUAC1 channel	Yes	Imes et al., 2013
*A. thaliana*	Phosphorylation	OST1 protein kinase	K^+^ inward channel	Yes	Sato et al., 2009
*A. thaliana*	Phosphorylation	OST1 protein kinase	PIP 2;1 aquaporin	Yes	Grondin et al., 2015
*A. thaliana*	Phosphorylation	FLS2	BAK1	No	Schulze et al., 2010
*A. thaliana*	Phosphorylation	BAK1	FLS2	No	Schulze et al., 2010
*A. thaliana*	Phosphorylation	BAK1	BIK1	No	Lin et al., 2014a
*A. thaliana*	Phosphorylation	BIK1	RBOH D	No	Li et al., 2014
*A. thaliana*	Phosphorylation	CPK5	RBOH D	Yes	Dubiella et al., 2013
*A. thaliana*	Phosphorylation	BIK1	MPK3	Yes	Montillet et al., 2013
*A. thaliana*	Phosphorylation	BIK1	MPK6	Yes	Montillet et al., 2013
*A. thaliana*	Phosphorylation	RPM1-Induced kinase	RIN4	Yes	Lee et al., 2015
*A. thaliana*	Nitrosylation	NO	OST1	Yes	Wang et al., 2015a
*B. napus*	Redox	ROS	BnSnRK2.4	Yes	Zhu et al., 2014
*B. napus*	Redox	ROS	IPMDH1	Yes	Zhu et al., 2014
*A. thaliana*	Nitrosylation	NO	NPR1	No	Mou et al., 2003; Waszczak et al., 2015
*A. thaliana*	Nitrosylation	NO	TGA transcriptional factor	No	Lindermayr et al., 2010
*A. thaliana*	Nitrosylation	NO	RBOH D	No	Yun et al., 2011
*A. thaliana*	Nitrosylation	NO	SABP 3	No	Wang et al., 2009

*Please refer to the text for abbreviations*.

Evidence also indicates there are protein kinases that function in parallel to OST1. For example, CPK6 (Brandt et al., 2012b), CPK21/23 (Geiger et al., 2010), and Guard cell Hydrogen peroxide-Resistant 1 (GHR1) phosphorylate SLAC1 to activate the anion channels upon ABA treatment, forming a redundant signaling pathway (Table 1). However, detailed characterizations using a loss- and gain-of-function approach imply that OST1 is still the central node and limiting factor in ABA guard cell signaling (Acharya et al., 2013). In addition to drought stress, protein phosphorylation may also play a role in stomatal movement in response to other abiotic stresses. For example, mutants of *MPK9* and *MPK12* are partially impaired in cold-induced stomatal closure, suggesting that the two kinases may function in a cold signaling pathway (Jammes et al., 2009). Further studies are needed to identify the MPK9 and MPK12 targets, and their roles in guard cell cold stress signaling cascade.

### Protein phosphorylation in guard cells under biotic stress

Stomatal pores, as the major gate of pathogen entry, constitute the first line of defense to prevent infection of the plant body by efficient stomatal closure. This process is initiated with the detection of the conserved PAMPs by various immune receptors. One of the best characterized interactions is the flagellin N-terminal 22 amino acid peptide (flg22) and the PRR Flagellin-Sensitive 2 (FLS2) and co-receptor Brassinosteroid insensitive 1-Associated Kinase 1 (BAK1) (Chinchilla et al., 2007; Sun et al., 2013). Using genetic and biochemical approaches, Schulze et al. (2010) showed that phosphorylation of FLS2 and BAK1 were detected within 15 s after flg22 treatment of *Arabidopsis* plants, and the kinase activity of BAK1 was required for flg22 perception (Table 1). Although there is no study specific for guard cells showing that FLS2 and BAK1 are phosphorylated after flg22 perception, the same events are likely to occur in the guard cells. It is known that FLS2 plays an important role in flg22-induced stomatal closure, since stomata in Arabidopsis *fls2* mutant are completely impaired by flg22 carrying pathogen *Pst*. DC3000 (Zeng and He, 2010). Genetics and biochemical approaches showed that activation of FLS2 and BAK1 in *Arabidopsis* plants promote formation of the receptor complex with the botrytis-induced kinase 1 (BIK1, Table 1). BIK1 phosphorylates RBOH D (Table 1), which directly modulates stomatal closure in response to flg22, as *rboh D* mutant and Arabidopsis carrying RBOH D^S39A, S343A, S347A^ exhibited completely impaired stomatal closure under flg22 treatment (Li et al., 2014). Interestingly, *Arabidopsis* RBOH D was also shown to be phosphorylated by CPK5 upon flg22 treatment (Dubiella et al., 2013, Table 1). In addition to RBOH D activation, flg22-induced FLS2 receptor complex also activates MPK3 and MPK6 to induce stomatal closure (Montillet et al., 2013). Thus, phosphorylation is an essential and common mechanism in pattern triggered immunity (PTI) responses. Downstream of PTI signaling includes regulation of K^+^ channels, turgor decrease in guard cells, and closure of the stomatal pores to prevent pathogen entry (Zhang et al., 2008; Zeng et al., 2010).

Successful pathogens deliver effector proteins into the plant cells to overcome PTI, and the effectors trigger the second layer of plant immunity called effector triggered immunity (ETI). For example, the bacterial effectors AvrB can be recognized by the plant immune receptor Resistance to *Pseudomonas syringae* pv Maculicola 1 (RPM1). Recognition of AvrB by RPM1 causes phosphorylation of RPM1-Interacting Protein4 (RIN4) by RPM1-Induced Protein Kinase (RIPK, Table 1). Recently, Lee et al. (2015) showed that RIN4^T21D∕S160D∕T166D^, a mutant with three phosphorylation sites changed to phosphorylation mimic aspartate residues, rendered Arabidopsis plants to exhibit large stomatal apertures and decreased resistance to *P. syringae*. This exemplifies how an effector protein facilitates pathogen infection by modulating host cell protein phosphorylation events.

### Current questions in guard cell protein phosphorylation research

As more aspects of phosphorylation in stomatal movement have been revealed, more questions have also been raised. The findings of OST1, as a central player in the core ABA pathway, open doors for questions such as how the activity of this key modulator is controlled. Is it activated by autophosphorylation or by an upstream kinase? How is OST1 dephosphorylated? Recently, Casein Kinase 2 (CK2) has been shown as a negative regulator of OST1 by increasing the binding of CK2-phosphorylated OST1 to PP2C (Vilela et al., 2015). With many key kinases identified in guard cells including SnRKs, CPKs, and MPKs, how are these kinase pathways crosstalk to minimize redundancy, and how is the signal specificity determined? What are the target proteins involved in stomatal movement? With the development of kinase substrates screening (Umezawa et al., 2013; Wang et al., 2013) and techniques in live-cell phosphorylation detection (Hayashi et al., 2011), more studies are forthcoming toward better understanding of the phosphorylation-mediated stomatal movement at high spatial and temporal resolution. In addition, since phosphorylation is essential in both ABA and flg22 triggered stomatal closure, what are the convergent nodes and edges? This question is still under debate. One study showed that the flg22 response was independent of ABA signaling (Montillet et al., 2013), while another study indicated that flg22 induced stomatal closure was impaired in the *ost1* mutant (Guzel Deger et al., 2015). It should be noted that in the first study 10 times more flg22 was used to cause stomatal movement in the *ost1* mutant. Therefore, it is likely that both ABA-dependent and independent pathways are functional. In addition, different protein kinases may be involved in different pathways. For example, MPK3 and MPK6 were shown to be important players in flg22 triggered stomatal closure (Montillet et al., 2013), while MPK9 and MPK12 play critical roles in the guard cell ABA and cold stress signaling (Jammes et al., 2009), as well as yeast elicitor signaling (Salam et al., 2013). Moreover, both protein kinases and phosphatases control the dynamics of protein phosphorylation in guard cell signaling. However, only a few phosphatases have been identified in guard cells (Tseng and Briggs, 2010; Sun et al., 2012; Takemiya et al., 2013), and their interactions with key signaling proteins remaining largely elusive.

## Redox-dependent PTMs in stomatal functions

As with protein phosphorylation and other PTMs, redox-dependent PTMs may function as molecular switches to turn on or off signaling processes in plant response to abiotic and biotic stresses. Thiol is a nucleophile that when exposed to oxidative stress, undergoes reversible inter- and intra-molecular disulfide bond formation, nitrosylation, glutathionylation, sulfenic acid and sulfinic acid modification, and irreversible sulfonic acid modification. Additionally, the high pKa values of protein cysteines make these residues highly responsive to small redox perturbation (Spoel and Loake, 2011). The production of ROS and NO is a common event during stomatal closure (Xie et al., 2014). The ROS and NO can serve as signaling molecules by modifying the reactive protein thiol groups. Here we focus on recent progress on the roles of redox-dependent cysteine PTMs in stomatal movement under abiotic and biotic stresses.

### Redox PTMs in guard cells under abiotic stress

As described in the previous section, under drought stress ABA-induced stomatal closure is associated with an increase in NO and ROS production in guard cells (Zhang et al., 2001; Neill et al., 2008). The ROS production is catalyzed mainly by two types of enzymes, the plasma membrane NADPH oxidases, and the cell wall peroxidases (Sharma et al., 2012). Other ROS-generating enzymes, such as apoplastic amine oxidases and oxalate oxidases, may also be involved in ROS production leading to stomatal closure (Tripathy and Oelmüller, 2012). The NADPH oxidases are regulated by direct binding of Ca^2+^ (Kadota et al., 2015), phosphatidic acid (Zhang et al., 2009), Rac GTPases (Wong et al., 2007), and via phosphorylation by OST1 (Sirichandra et al., 2009), CDPKs (Kadota et al., 2015), and BIK1 (Kadota et al., 2014). Consequently, NADPH oxidase may integrate multiple upstream signaling events to promote stomatal closure. NO is produced by the nitrite-dependent nitrate reductase pathway (Desikan et al., 2002) and a nitric oxide associated 1 (NOA1) protein-dependent pathway (Lozano-Juste and León, 2010). It is important to note that the NOA1 is not a NO synthase (Moreau et al., 2008).

Although the essential function of ROS and NO in stomatal closure has been widely accepted, little is known about the underlying molecular mechanisms, by which they achieve the PTM regulation in guard cells. Thus, direct evidence for thiol-based redox regulation under stress conditions and a link between protein redox regulation and stomatal movement need to be established. A recent study showed that NO resulting from the ABA signaling caused S-nitrosylation of OST1 at the cysteine residue (Cys137) close to the kinase catalytic site (Table 1), and the PTM abolished the kinase activity (Figure 1B). This represents an interesting negative feedback mechanism by which ABA-induced NO helps to desensitize ABA signaling. Additionally, the authors showed that the Cys137 is evolutionarily conserved in some AMPK/SNF1-related kinases and glycogen synthase kinase 3/SHAGGY-like kinases (SKs) in plants, yeast and mammals, and the S-nitrosylation-mediated inhibition may be a general regulatory mechanism (Wang et al., 2015a). This example also highlighted how redox changes regulate protein kinase phosphorylation and signaling cascade in stomatal movement.

In a redox-proteomics study, Zhu et al. (2014) identified 65 and 118 potential redox responsive proteins in ABA and MeJA treated *Brassica napus* guard cells, respectively. The authors demonstrated that most of the proteins belong to functional groups such as energy, stress and defense, and metabolism. In addition, osmotic stress-activated protein kinase (BnSnRK2) and isopropylmalate dehydrogenase (IPMDH) were confirmed to be redox regulated and involved in stomatal movement (Table 1). These findings demonstrate the utility of redox-proteomics in discovering uncharacterized redox proteins and their roles in stomatal movement. Although some proteins have been identified to be redox regulated, their functions in regulating stomatal movement are still to be fully characterized.

### Redox PTMs in guard cells under biotic stress

Pathogen perception initiates a signal transduction cascade including ROS and NO production, increase in Ca^2+^ influx, alkalization of the extracellular space, activation of MAPK, CDPK, salicylic acid (SA) pathway, and synthesis of ethylene (Arnaud and Hwang, 2015). The ROS and NO generated under biotic stresses are known to act as antimicrobial compounds. ROS are also known to be involved in cell wall cross-linking and blockage of pathogen infection (Torres et al., 2006). Furthermore, they play important signaling roles, e.g., in redox PTM of essential proteins in plant defense (Agurla et al., 2014). Methionine and cysteine residues of certain proteins are sensitive to H_2_O_2_ and NO (Hoshi and Heinemann, 2001). The sensitivity of the residues depends on the protein structure, neighboring residues, and solvent accessibility (Roos et al., 2013). H_2_O_2_ can react with a cysteine thiolate forming intra- or inter- disulfide bonds, sulfenic acid (-SOH), sulfinic acid (-SO_2_H), and sulfonic (-SO_3_H) acid (Dalle-Donne et al., 2006). NO can covalent bind to a cysteine thiol through S-nitrosylation.

Although redox-dependent PTMs in biotic stresses is an emerging field, there are some examples showing the redox regulation of proteins in guard cells. In plant defense, Nonexpresser of PR gene 1 (NPR1) is one of a limited number of examples of protein redox regulation. NPR1 was detected primarily in the cytoplasm and nuclei of guard cells (Kinkema et al., 2000). Under normal conditions, NPR1 is retained in the cytoplasm as inactive disulfide-bonded oligomers, which is promoted by the S-nitrosylation at cysteine 156 (Table 1). In the presence of pathogen, an increase in SA mediates cellular redox changes, leading to thioredoxin-mediated reduction of the NPR1 oligomer to monomeric forms, which are then transported into the nucleus to activate plant immune processes (Mou et al., 2003; Waszczak et al., 2015). In the nucleus, SA mediated redox change causes de-nitrosylation and reduction of disulfide bonds in TGA transcriptional factors (Table 1) so that they can form an active transcriptional complex with NPR1 to turn on pathogenesis related (PR) genes (Lindermayr et al., 2010, Figure 2), and NPR1 is then phosphorylated and ubiquitinylated for degradation (Waszczak et al., 2015). Although protein redox regulation is not well studied in plant innate immunity, it is clear from the above example that modification of cysteine thiols can alter protein activity, function, and redox crosstalk with other modifications.

**Figure 2:**
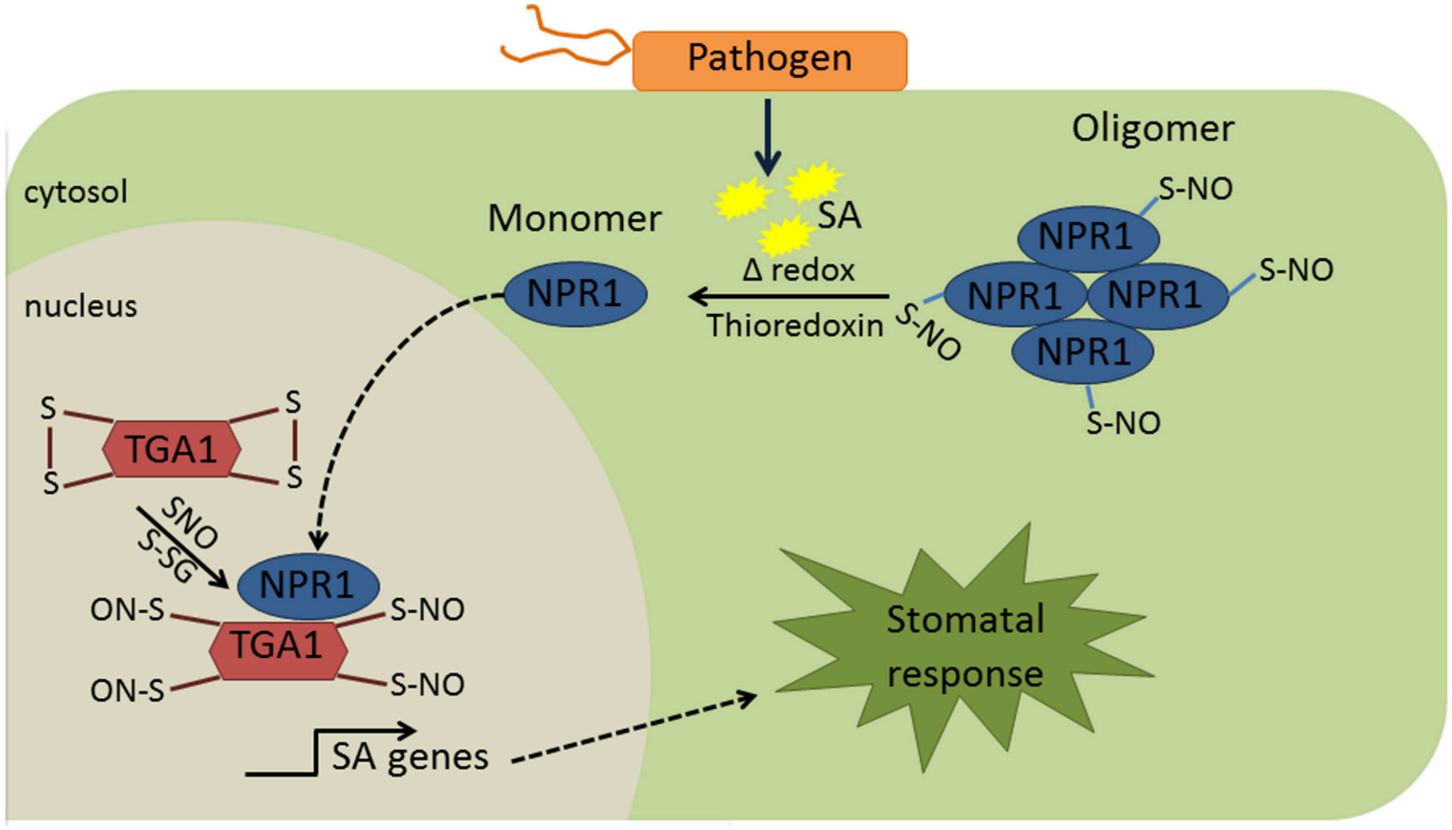
**Redox regulation of NPR1 and TGA1**. Under normal conditions, NPR1 is retained in the cytosol as an oligomer. S-nitrosylation of NPR1 is known to promote NPR1 oligomerization. In the presence of pathogen, production of SA promotes cellular redox changes, which will contribute to reduction of the NPR1 oligomer to monomeric form. Monomeric form of NPR1 moves to the nucleus and binds to TGA1 that was nitrosylated due to cellular redox changes mediated by SA. The complex NPR1-TGA1 turns on the transcription of PR genes. Although this mechanism was not directly elucidated in the guard cells, it is likely to be the case since NPR1 was primarily in the cytosol and nucleus of guard cells (Kinkema et al., 2000). SA, salicylic acid; NPR1, nonexpresser of PR gene 1; TGA1, teosine glume architecture 1; SNO, S-nitrosylation; S-GS, S-glutathionylation.

Yun et al. (2011) demonstrated a NO biphasic control in pathogen triggered cell death. At the initial stage of pathogen infection, S-nitrosothiol (SNO) accumulation leads to accelerated cell death. Conversely, constitutively high SNO levels promote decreased cell death through S-nitrosylation of RBOH D (Table 1), leading to reduction in its activity and oxidative stress. This differential regulation seems important in fine tuning the extent of cell death under conditions of abiotic and biotic stresses, since both cause increases of NO levels. At a certain level of NO concentration, the signaling components of stomatal movement and plant response may be unresponsive or irreversibly regulated with detrimental effects on stress acclimation. During the NO burst, NO also promotes S-nitrosylation of an Arabidopsis SA-binding protein 3 (AtSABP3) at Cys280 (Table 1). The S-nitrosylation suppresses both SA binding, and its chloroplast carbonic anhydrase activity (Wang et al., 2009). Interestingly, in tobacco SABP3 showed antioxidant activity and plays a role in the hypersensitive defense response (Slaymaker et al., 2002). Although the role of SABP3 nitrosylation in stomatal closure in response to biotic stresses has not been studied, it may play a role in stomatal movement signaling as SA is known to promote stomatal closure (Khokon et al., 2011). The examples here demonstrated the great potential of redox regulation in stomatal movement in response to biotic stresses. The development of redox proteomics technologies such as the cysTMTRAQ (Parker et al., 2015) and application of genetics, biochemistry, metabolism, and bioinformatics tools would accelerate the discovery and characterization of redox-dependent PTMs of proteins and their roles in stomatal signaling and plant immunity.

## Concluding remarks

Regulation of the size of the stomatal aperture is an essential mechanism in plants for optimizing the efficiency of water usage and photosynthesis. Stomatal movement through dynamic changes of the turgor of guard cells represents the output of integration of environmental signals with cellular signal transduction networks. Perception of abiotic and/or biotic stress signals triggers activation of signal transduction cascade, leading to rapid guard cell responses, which are known to be regulated by PTMs (e.g., protein phosphorylation and redox modification) of key players in the complex guard cell signaling networks. Over the past years, improvement and development of new tools in proteomics and MS have enabled the identification of PTMs of proteins involved in stomatal movement. In fact, LC-MS/MS based PTMomics technologies have become indispensable in identification and mapping of novel protein phosphorylation and redox modification sites. Additional sample preparation techniques, such as PTM enrichment and specific isotope labeling have greatly helped the detection and quantification of protein phosphorylation and redox changes, and thereby the understanding of PTM-controlled signaling pathways. The past decade has seen exciting discoveries in ABA and bacterial pathogen-triggered PTMs, especially phosphorylation and redox modification. Despite of current progress, guard cell PTMomics is still in its infancy and many aspects of protein level regulations remain elusive. For example, the crosstalk among different PTMs, and PTMs involved in regulating stomatal movement in response to other environmental factors are largely unknown. The fast advancement of proteomics technologies, together with genetics, molecular biology, biochemistry, and bioinformatics tools will accelerate the discovery and characterization of novel PTMs, and provide new insights into the complex protein phosphorylation and redox regulatory networks in guard cell signal transduction.

## Author contributions

KB drafted the manuscript with assistance from TZ. KB drew the figures. TZ focused on phosphorylation sections. SC provided guidance, edited and finalized the manuscript.

### Conflict of interest statement

The authors declare that the research was conducted in the absence of any commercial or financial relationships that could be construed as a potential conflict of interest.
